# FRET-Based System for Probing Protein-Protein Interactions between σ^R^ and RsrA from *Streptomyces Coelicolor* in Response to the Redox Environment

**DOI:** 10.1371/journal.pone.0092330

**Published:** 2014-03-20

**Authors:** Zi-Han Wei, Huan Chen, Chang Zhang, Bang-Ce Ye

**Affiliations:** State Key Laboratory of Bioreactor Engineering, East China University of Science and Technology, Shanghai, China; Instituto de Biociencias - Universidade de São Paulo, Brazil

## Abstract

Protein-protein interactions between sigma factor σ^R^ and its corresponding zinc-binding anti-sigma (ZAS) protein RsrA trigger the thioredoxin system for maintaining cellular redox homeostasis in *S. coelicolor.* RsrA bound to zinc associates with σ^R^, inhibiting its transcriptional activity in a reducing environment. During disulfide stress it forms intramolecular disulfide bonds, leading to zinc release and dissociation from σ^R^, which initiates transcription to produce reductase and thioredoxin. We designed a fluorescence resonance energy transfer (FRET) based system for monitoring protein-protein interactions between σ^R^ and RsrA to further understand how this redox switch regulates the thioredoxin system in *S. coelicolor* in response to its redox environment, especially various reactive oxygen species (ROS) derived from different metabolic pathways, and clarify the different response mechanisms between Zn-RsrA and apo-RsrA. By the use of the FRET approach described here, we showed that zinc protected thiols in RsrA and causes the σ^R^-RsrA complex to form a more compact structure. This system was also utilized to detect changes in redox status induced by ROS and diamide in real time in *E. coli* cells.

## Introduction

Protein-protein interactions are central to complex cellular functions and the regulation of protein activity is a major factor in the cellular response to a changing environment [1]. Disulfide bond formation due to thiol oxidation in proteins, for example, alters their properties of interaction and may trigger activation of the anti-oxidative stress system in cells [2]. Regulatory systems involving thiol oxidation, such as NF-kB in mammalian cells [3], [4], OxyR in *E*. *coli* [5], and redox-sensitive cofactors like FAD [6] form disulfide bonds to avoid deleterious damage caused by reactive oxygen species (ROS) [7]. ROS-induced thiol oxidation, on the other hand, can be reduced by conserved oxidoreductases such as thioredoxin and glutaredoxin in a regulatory feedback loop [8], [9]. The cytoplasm of all organisms is a highly reducing environment in which protein cysteines are maintained in their reduced thiol (-SH) state. Organisms have evolved regulatory systems to sense and respond to peroxide stress (ROS) and disulfide stress (such as the thiol-specific oxidant diamide) in order to maintain cellular redox homeostasis [10]. In bacteria, many kinds of regulatory proteins that govern the oxidative stress response have been investigated, such as peroxide-sensing OxyR (disulfide bond formation between Cys199 and Cys208 in the presence of H_2_O_2_) [5], peroxide-sensing PerR (iron-catalyzed-oxidation of His37 in the presence of H_2_O_2_) [11], and organic peroxide-sensing OhrR (oxidation of Cys15 and formation of Cys-SOH or Cys-SO_2_H derivatives in the presence of organic peroxides) [12]. In the soil-dwelling Gram-positive bacterium *Streptomyces coelicolor,* the model representative of antibiotic-producing actinomycetes, the mechanism for regulating the thioredoxin system during oxidative stress is mainly based on reversible disulfide bond formation in the zinc-binding anti-sigma (ZAS) protein RsrA [13]–[15].

The interaction between an extracytoplasmic function (ECF)-type sigma factor σ^R^ and RsrA regulates the thioredoxin system in *S. coelicolor*. The ZAS protein RsrA modulates the activity of σ^R^ as a σ^R^–specific anti-sigma factor [16], [17]. It has been documented that in the reducing environment of the cytoplasm, RsrA binds together with a zinc ion to σ^R^ to form a stable 1∶1 complex, preventing it from activating transcription. Disulfide stress induces intramolecular disulfide bond formation between Cys11 and Cys44 in RsrA, leading to release of zinc and dissociation of σ^R^ [13], [18]. Free σ^R^ initiates transcription to produce reductase and thioredoxin and auto-regulates transcription of *sigR* to produce more σ^R^ itself. Thus σ^R^ and RsrA (binding with zinc) together form the redox switch σ^R^-RsrA. In *S. coelicolor,* RsrA-σ^R^ controls a regulon of approximately 60 genes that help the bacterium survive disulfide stress, such as *trxBA* and *trxC* that encode thioredoxin and thioredoxin reductase [9], [14]. The newly expressed thioredoxins reduce the disulfide bonds in RsrA to free thiols, leading to immediate rebinding of zinc and inhibition of free σ^R^ until redox homeostasis is re-established. Interestingly, zinc is not necessary for the interaction between σ^R^ and RsrA, though it plays an important role in coordinating the conformation of RsrA during disulfide stress [18]. Two models have been presented for the single zinc site in RsrA. One model considers Cys3, His7, Cys41, and Cys44 to be the metal ligands [13] while the other considers Cys11, Cys41, Cys44 and His37, which fits the conserved ZAS motif residues (H37xxxC41xxC44) to be the metal ligands in RsrA [8].

Fluorescence resonance energy transfer (FRET) is an optical technique whereby the excited energy state of a fluorescent donor molecule is transferred to a ground state of an acceptor molecule by means of long-range resonance coupling between the donor and acceptor transition dipoles [19]–[21]. FRET is widely employed in developing genetically encoded biosensors to better understand the spatiotemporal regulation of various cellular processes [22], [23]. To take advantage of real-time measurement techniques, FRET-based interaction assays hold great promise for use in monitoring protein-protein interactions and protein conformational changes in live biological samples [24], [25]. Relying on the protein-protein interaction between σ^R^ and RsrA, it is possible to utilize FRET to confirm the oxidative stress response mechanism by recombining a fluorophore with a target protein. Therefore, yellow fluorescent protein (excitation at 440 nm, emission at 478 nm) was linked to σ^R^ as an acceptor, creating the *sigR*-*yfp*-pET28a (+) protein (SYP), and cyan fluorescent protein (excitation at 440 nm, emission at 528 nm) was linked to RsrA as a donor, creating the *rsrA*-*cfp*-pET28a (+) protein (RCP). Theoretically, the donor RCP binds to the acceptor SYP, emitting a FRET signal in a reducing environment; then, RCP separates from SYP, turning off the FRET signal, during disulfide stress.

In this study, we developed a pair of novel FRET-based and genetically encoded probes to investigate the interaction between σ^R^ and RsrA in response to redox environment. The activity of the probes was tested by biolayer interferometry. It showed the same function as the original redox switch σ^R^-RsrA without fluorescent proteins. The kinetic parameters of binding between RsrA and σ^R^ were then obtained by the probes. From the data, the different disulfide stress responses of apo-RsrA and Zn-RsrA suggest the redox switch is triggered in two steps, firstly zinc release, and then complete dissociation of apo-RsrA. Moreover, we detected the sensitivities of the σ^R^-RsrA complex in response to various ROS derived from different metabolic pathways for further understanding of its relevant regulation mechanism within the whole network of metabolism. The probes were also used to monitor changes in redox status induced by ROS and diamide in real time in *E. coli*.

## Materials and Methods

### Plasmid construction

The plasmids encoding the His-tagged SYP and RCP fusion proteins were constructed in two steps. In the first step, the *rsrA* or *sigR* were amplified by polymerase chain reaction (PCR) from the *S. coelicolor* M145 genome using specific oligonucleotides containing two restriction sites (Table 1). The *rsrA* PCR fragments were digested with Nco I and EcoR I and ligated into pET28a (+) that was predigested with Nco I and EcoR I, yielding *rsrA*-pET28a (+). The PCR fragments of *sigR* were digested with Nco I and Hind III and ligated into pET28a (+) that was predigested with Nco I and Hind III, yielding *sigR*-pET28a (+). In the second step, the *yfp* and *cfp* genes were amplified by PCR from pEcfp-N1 and pEyfp-N1, respectively, using specific oligonucleotides containing two restriction sites (Table 1). The *cfp* PCR fragments were digested with EcoR I and Hind III and ligated into *rsrA*-pET28a (+) that was predigested with EcoR I and Hind III, yielding *rsrA*-*cfp*-pET28a (+). The *yfp* PCR fragments were digested with Hind III and Xho I and ligated into *sigR*-pET28a (+) that was predigested with Hind III and Xho I, yielding *sigR*-*yfp*-pET28a (+).

**Table 1:** Sequences of PCR primers for construction of corresponding plasmids.

Sequence	Oligonucleotides (5'-3')	Restriction site
rsrAA	CATGCCATGGGCAGCTGCGGAGAGCCG	NcoI
rsrAB	CCGGAATTCGGACTCCTGCGGGGCCG	EcoRI
sigRC	CATGCCATGGGCACTGGGACCGACGCA	NcoI
sigRD	GGGAAGCTTTGACCCCGAGCCTTTCG	HindIII
cfpA	CGGAATTCGGAGGAAGCATGGTGAGCAAGGGCG	EcoRI
cfpB	CCCAAGCTTCTTGTACAGCTCGTCCATGC	HindIII
yfpC	CCCAAGCTTGGAGGAAGCATGGTGAGCAAGGGCGA	HindIII
yfpD	CCGCTCGAGCTTGTACAGCTCGTCCATGCC	XhoI

### Expression and purification of the FRET-based recombinants

*Escherichia coli* BL21 (DE3) cells were transformed with the constructed plasmids encoding RsrA, σ^R^, RCP, SYP, Cyan Fluorescent Protein (CFP) and Yellow Fluorescent Protein (YFP). Cultures of the transformants grown in LB broth containing 50 μg/ml kanamycin at 37°C were treated with 1 mM isopropyl-β-D-thiogalactopyranoside (IPTG) C, once they reached an OD_600_ of 0.5, for 10 h at 30°C to induce expression of the RCP or SYP protein. Cells were resuspended in PBS (137 mM NaCl, 2.7 mM KCl, 10 mM Na_2_HPO_4_ and 2 mM KH_2_PO_4_, pH 8.0) and sonicated in four cycles of 30 s on ice using the microtip of the Sonicator (Qsonica 500, Newtown, CT) ultrasonic processor. The cell extract was centrifuged (Sigma 3k15, Germany) for 30 min at 4500 rpm and 4°C to remove cell debris. His-tagged proteins were purified on Ni-nitrilotriacetic acid (Ni-NTA) agarose columns on AKTA-explorer (GE). Proteins were quantified by Lowry’s method with bovine serum albumin (BSA) used as the standard. Purity was judged by non-reducing Coomassie Blue staining of SDS-polyacrylamide (12.5%) gels. To remove imidazole and the extraction buffer, the protein solution was dialyzed against binding buffer (0.01 mM EDTA, 20% glycerol, 10 mM MgCl_2_, 40 mM Tris, pH 8.0). Proteins were sealed with paraffin oil and stored at 4°C until the assays.

### *In vitro* FRET-based detection of protein-protein interaction

Proper concentrations of samples (diluted with binding buffer if necessary) were prepared to fit the detection range of the fluorescence plate reader (Bio-Tek Instrument, Winooski, USA) and 0.5 μM RCP and 0.5 μM SYP were prepared for assays. All fluorescence based assays were undertaken in a 384 well microplate reader and kept in a reduced environment by ventilating nitrogen gas. Fluorescent spectra were scanned between wavelengths 460 nm and 600 nm at an excitation of 440 nm. Before the scan the samples were incubated in binding buffer for 30 minutes at 30°C. Titration and over time assays were detected at the endpoint of the fluorescent emission peak, 478 nm for RCP or CFP and 528 nm for SYP or YFP respectively, with an excitation of 440 nm. Reading procedures for proper time course were designed by software Gen 5 1.09. For titration detections, plates were read approximately 7 s after addition. The FRET ratio was defined as the fluorescence emission at 528 nm divided by the emission at 478 nm. One millimolar H_2_O_2_ or 2.5 mM DTT as final concentrations were added rapidly to samples in corresponding assays. In pH effect assays, 0.5 μM RCP was incubated with 0.5 μM SYP for 30 min before fluorescence detection for each pH group, and the pH of the binding buffer was modulated with aqueous 1 mM HCl. In FRET-based binding measurement increasing concentrations of purified SYP (from 0.1 nM to 0.85 μM) were detected at 528 nm endpoint with and without the existence of RCP in determined final concentration. RCP is prepared in binding buffer with addition of 2.5 mM DTT to ensure fully reduced RCP. FRET efficiency (E), defined as the probability of energy transfer per donor excitation event and can be obtained by measuring the intensity of donor emission in the absence (F_d_) and presence (F_da_) of an acceptor as the following equation [25]:
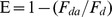



The obtained FRET efficiency values were fitted to a single, single-site binding curve as the following equation [26]:
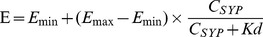



E_max_ and E_min_ represent the maximum and minimum FRET efficiency, respectively, and C_SYP_ represents the concentration of the SYP ligand. Thus, the difference between the values of E_max_ and E_min_ represents the FRET efficiency at a saturating concentration of SYP. In zinc function assay, purified proteins (including RCP, SYP, YFP and CFP) were digested by thrombin to remove their N-terminal histidine tag. Histidine residues were separated by dialysis against binding buffer with additional 2.5 mM DTT, which contains 5 mM EDTA to remove possible zinc in RCP during protein expression and purification. Increasing concentrations of ZnSO_4_ or CaCl_2_ were added into a mixture of SYP and RCP at the same concentrations. The FRET ratio change was defined as the FRET ratio (emission 528 nm/478 nm) after the addition of Zn^2+^ or Ca^2+^, subtracting the FRET ratio of the original sample. Then 500 μM (as final concentration) of ZnSO_4_ was selected for further *in vitro* assays. The same assays were performed on CFP and SYP as controls. Then DTT in samples was removed by dialysis in nitrogen saturated binding buffer. The fluorescent spectra of both the zinc containing sample and the apo-sample were scanned respectively before and after the addition of 1 mM diamide. Independent measurements were repeated three times. All the raw data in this study were processed into graphs using GraphPad Prism software unless specified otherwise.

### Circular dichroism spectroscopy

Circular dichroism spectra were obtained on a CD spectrophotometer (Applied Photophysics, UK) using a 10 mm cuvette equilibrated at 30°C. All the samples were desalted and the buffer exchanged into PBS (pH 8.0) through a Zeba™ Desalt Spin Column (Thermo Scientific). One millimolar H_2_O_2_ and 2.5 mM DTT as final concentrations were incubated in the corresponding samples during equilibration for 20 minutes. Sample concentrations were diluted to 0.15 μM. The CD spectra were collected between 190 nm and 280 nm, and 3 repeat scans were averaged. Parameters of secondary structure were calculated by the CD Spectra Deconvolution Program CDNN version 2.1.

### Kinetic binding measurements by biolayer interferometry

Binding kinetics and affinities between RsrA, σ^R^, SYP and RCP were measured by biolayer interferometry (Octet QK^e^, Fortebio, USA). Kinetic buffer (binding buffer, 1 mg/ml BSA, and 0.02% Tween 20) was used for binding experiments, step wash, association and dissociation. Prior to immobilization on Streptavidin (SA) biosensor tips (Fortebio), σ^R^ and SYP were desalted and buffer exchanged into PBS through a Zeba™ Desalt Spin Column and biotinylized for 1h respectively. Excess biotin was removed by the desalting column. Biotinylated ligands (σ^R^ or SYP ligand at 0.5 μM in PBS) were captured by biosensors during 5 min incubation, followed by a 5 min wash in kinetic buffer [27], [28]. Four increasing concentrations (2-fold serial dilutions starting at 200 nM) of RsrA or RCP as analyte were equilibrated in kinetic buffer. The association and dissociation binding kinetics for σ^R^ ligand and RsrA, σ^R^ ligand and RCP, SYP ligand and RsrA, and SYP ligand and RCP were measured for 14 min each. Binding affinities were independent of which protein was immobilized. Binding of σ^R^ ligand and CFP or YFP, and SYP ligand and CFP or YFP were measured as references, which were then subtracted during the data analysis. The binding kinetics in each group was measured and evaluated by Fortebio Data Analysis software 7.0 using a 1∶1 binding model to fit the curves.

### Preparation of ROS reagents

The reagents for dose-response assays were prepared in stock solutions or generated during operation. The concentration of H_2_O_2_ was determined based on the molar extinction coefficient at 240 nm (43.6 M^−1^cm^−1^) [29] by appropriate dilutions in double distilled water (dd H_2_O). The concentration of ^−^OCl was determined based on the molar extinction coefficient at 292 nm (350 M^−1^cm^−1^) [29] by diluting the NaOCl solution appropriately in 0.1 M aqueous NaOH. **^·^**OH was generated through Fenton reaction so that a final concentration of 1 mM H_2_O_2_ was added into samples, which included an appropriate concentration of FeSO_4_ [29]. O_2_ **^·^** ^−^ was generated through auto-oxidation of an appropriate concentration of pyrogallol in PBS (pH 8.2) for 4 minutes [30]. ONOO^−^ was generated through reaction between NaNO_2_ and H_2_O_2_ with acid (HNO_3_) diluted in water to an appropriate concentration. Excess H_2_O_2_ was removed by a column containing MnO_2_ particles. The concentration of ONOO^−^ was determined based on the molar extinction coefficient at 302 nm (1670 M^−1^cm^−1^) [30]. Diamide was prepared in stock solutions and diluted in water to appropriate concentrations.

### *In vivo* application as ROS biosensor in *E. coli*

The *syp* (*sigR*-*yfp*-pET28a (+)) was modified by inserting ampicillin resistant gene sequence in restriction site Sma I (4300 bp on pET28a (+)) within the original kanamycin resistant gene. The modified *syp* and *rcp* plasmids with different antibiotic marks (ampicillin and kanamycin respectively) were co-transferred into *E. coli* BL21 (DE3) with same concentration, and SYP and RCP expresson was induced by IPTG. *E. coli* cells were disposed by increasing concentrations of CaCl_2_ before H_2_O_2_ treatment (4 mM) detection and the optimized concentration was selected for further assays based on ΔFRET ratio values. The fluorescent emission endpoints of the samples at 478 nm and 528 nm were detected in serial wells in a 384 well microplate at an excitation of 440 nm. The samples were then incubated with the same concentration of ROS (100 μM prepared the same as in the *in vitro* assays) for 25 min at 30°C, and were detected again. For the time course assays, fluorescent emission endpoint at 478 nm and 528 nm of samples were detected over time for 5 minutes, and then 1 mM diamide or a proper concentration of various ROS whose concentration at half-maximal saturation of ΔFRET ratio in each curve of *in vitro* dose-response experiment (3.7 mM H_2_O_2_, 1.8 mM ^−^OCl, 45 μM O_2_ **^·^** ^−^, 70 μM ONOO^−^, 140 μM **^·^**OH) was added and the same fluorescent emission endpoint tested overtime for another 30 minutes. *E. coli* cells were suspended in PBS (pH 7.8) during the assays and buffer was added as the control of blank.

## Results

### Construction and Characterization of FRET-based probes for detecting the protein-protein interaction of σ^R^ and RsrA in response to the redox environment

**Figure 1:**
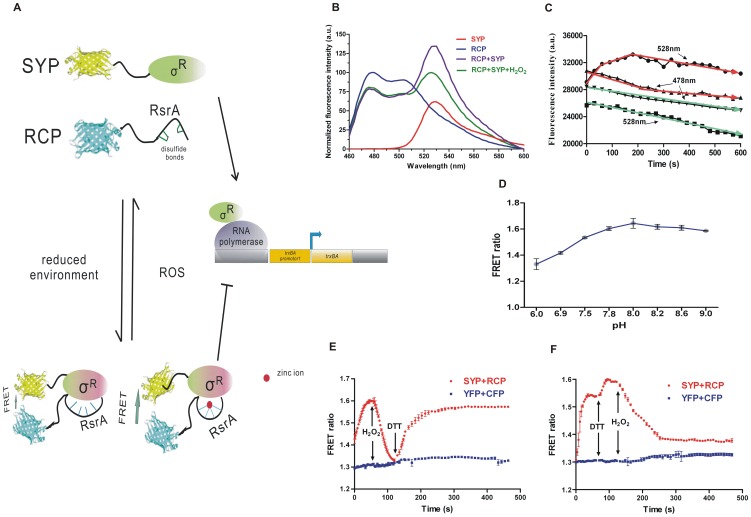
Characterization of FRET-based probe. (A) Schematic of FRET-based probes and redox response mechanism. (B) Fluorescent spectra of SYP and RCP, and mixture with or without H_2_O_2_. Fluorescent intensities with excitation at 440 nm were normalized to the initial value. (C) Time course of fluorescent emission adding SYP into RCP at wavelengths of 528 nm and 478 nm with 30 s interval. Detection of CFP added to YFP was performed as a control. (D) Effect of pH on fluorescent based probes. (E) and (F) Time course of FRET ratio of SYP and RCP incubation in response to redox environment in independent experiments with different order: H_2_O_2_ and DTT were added at indicative time points. RCP was added into the SYP solution at the start point of detection. The addition operation interrupted the measurement for approximate 7 s. YFP and CFP were detected as reference. Data represent the means ± SEM of duplicates of three independent measurements.

The σ^R^-RsrA complex, acting as a redox switch, controls the thioredoxin reducing system by a conformational change in RsrA in response to disulfide stress. We designed the FRET-based probes SYP and RCP by linking CFP with RsrA and YFP with σ^R^ to monitor the protein-protein interaction between RsrA and σ^R^ in response to the redox environment (Fig. 1A). A flexible glysine-glysine-serine linker between the fluorescent protein and the targeted protein has a positive effect on the efficiency of FRET and attenuates the effect of the fused protein on the conformation of the target protein [24], [31]. Binding of RsrA to σ^R^ leads to an appropriate change in distance between YFP and CFP. In a reducing environment, RCP binds to SYP to emit FRET signal, which would be diminished in an oxidizing environment or in response to a direct oxidant.

The emission spectra of SYP and RCP separately excited at 440 nm presented similarities to YFP and CFP, respectively, but a striking FRET signal (the emission peak at 478 nm decreases and the peak at 528 nm increases) appeared when SYP and RCP were incubated together (Fig. 1B). The fluorescent intensity of the spectra reflected the corresponding concentration of the sample, so that the 478 nm peak decreased for the mixture of SYP and RCP. Then, the addition of peroxide (H_2_O_2_) turned off the FRET signal, which indicates the dissociation of SYP and RCP derives from the anti-oxidative property of the σ^R^-RsrA complex. Therefore, we can use this pair of fluorescence probes to monitor the protein-protein interaction and how the disulfide stress response system initiates in *S. coelicolor* in real time.

When titration of RCP to SYP was determined *in vitro* over time, we found that equilibrium binding was reached within 5 minutes and the most active period appeared during the first minute (Fig. 1C). As for the genetically encoded fluorescence probes, the effect of pH during *in vitro* experiments must also be taken into consideration. We investigated the fluorescent emission ratio (528 nm/478 nm) of the same SYP and RCP samples at different pH in binding buffer. It was observed that lower pH (6.0) greatly quenched the FRET signal. The ratio was almost invariant from pH 7.5 to 8.5 (Fig. 1D), suggesting the fluorescence signal of the FRET system would not be affected by small pH fluctuations in a neutral or basic environment.

Then the binding of SYP and RCP and its response to the redox environment was determined over time to verify the process of initiating the anti-oxidative stress mechanism (Fig. 1E). The initial rise in the fluorescent protein YFP/CFP emission ratio (528 nm/478 nm) value indicated that the association reaction between SYP and RCP produces the FRET signal. At 60 s into the time course, a time point with a 3 s interval chosen based on the *in vitro* time course assay, H_2_O_2_ was added, leading to an obvious and immediate decrease in the ratio’s value. Then, at 120 s, addition of DTT caused the value of the ratio to increase again and reach saturation after approximately 300 s with a 10 s interval, but not back to the original level seen before addition of H_2_O_2,_ which was probably caused by additional air oxidation during the assay. The value of the ratio increased after adding DTT, indicating the reversible structural change in RsrA, as previous research has reported [17], [18]. DTT, acting as a protector of the RsrA structure, both neutralized the remaining H_2_O_2_ and partially reduced some of the disulfide bonds. When we changed the order of addition of H_2_O_2_ and DTT, an increase in the value of the ratio from 60 s to 120 s followed by a decrease was obtained as expected (Fig. 1F). However, the response range after adding H_2_O_2_ in Figure 1E is lower than that of Figure 1D, which probably results from the function of DTT. RsrA is very sensitive to air oxidation, so it explains why FRET ratio increased after addition of DTT, since fully reduced RCP would not be affected by additional DTT. In this case, the redox status of RsrA can also be readily detected by the change of FRET ratio. No obvious change in the FRET ratio was observed after adding H_2_O_2_ and DTT to YFP and CFP as controls.

### Functional equivalence of FRET-based recombinants (SYP and RCP) and original proteins (σ^R^ and RsrA)

**Figure 2:**
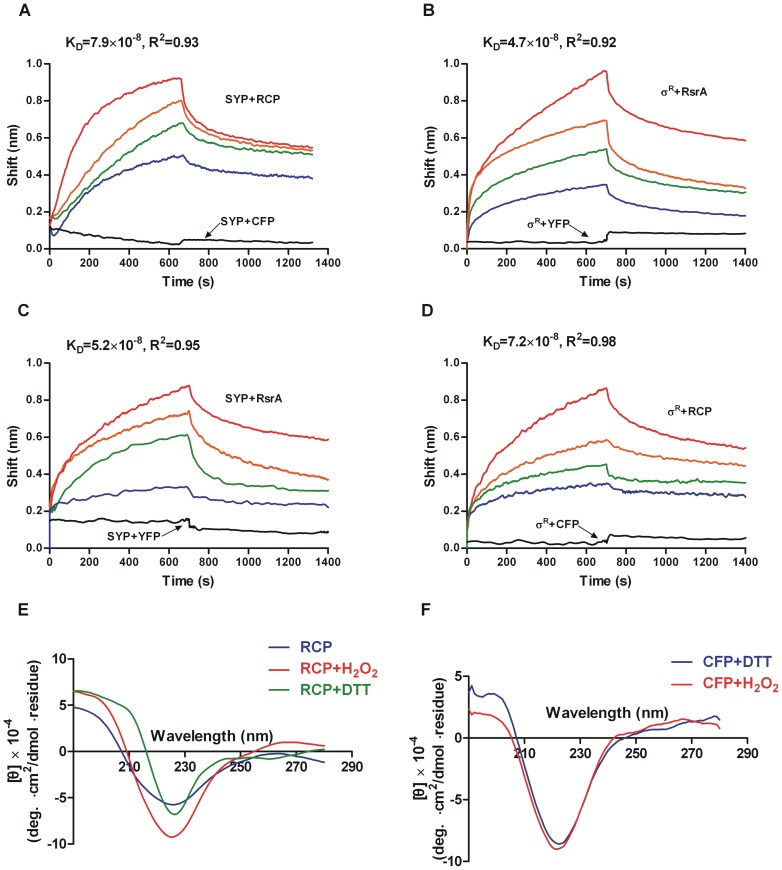
Function confirmation of the FRET-based probes by biolayer interferometry and Circular dichroism. Sensorgrams of SYP ligand on Streptavidin-coated biosensors binding to different concentrations of RCP (A) or RsrA (C), and σ^R^ ligand binding to different concentrations of RCP (B) or RsrA (D) are shown. Black lines show binding of ligands to fluorescence proteins as reference. The binding affinity parameter K_D_ is calculated and shown above binding curve for each measurement. R^2^ is the coefficient of determination estimating the goodness of a curve fit reported by Fortebio Data Analysis software version 7.0. Circular dichroism spectra of RCP in different redox environments were measured. (E**)** Secondary structure characterization (blue line) and conformational change of RCP in response to H_2_O_2_ (red line) and DTT (green line) after subtracting baseline of buffer are shown from 190 nm to 280 nm. (F) Conformational change of CFP in response to the redox environment (red line shows H_2_O_2_ and blue line shows DTT) as control.

Fluorescence resonance energy transfer from donor to acceptor can have many causes in genetically encoded probes in this study, for instance, a change in orientation or distance between the YFP and CFP fusions [25], [32]. Herein we used a non-fluorescence based method, biolayer interferometry (Octet QK^e^, *forte*bio, USA), to determine whether this pair of FRET-based probes, SYP and RCP, acts as the original proteins σ^R^ and RsrA. Either SYP or σ^R^ as a ligand was immobilized on SA sensors (Streptavidin-modified sensors) to bind RCP or RsrA as analytes, respectively (Fig. 2A-D). As expected, σ^R^ bound to either RsrA or RCP, and the same results were obtained with SYP. However, neither σ^R^ nor SYP bound to YFP and CFP as a reference, indicating the binding between SYP and RCP was due entirely to the σ^R^ and RsrA domains in the fusions. In each binding group, different concentrations of analyte were measured to evaluate the binding kinetics and affinities. Also as expected, the binding kinetics between the four groups were in the same order, which indicates the fused fluorescent protein has no obvious effect on the original protein and the recombinant SYP (or RCP) behaves exactly as the corresponding σ^R^ (or RsrA) protein.

The conformational change in RsrA is part of the oxidative stress response mechanism that leads to the release of free σ^R^ factor; therefore, the FRET on or off signal derives from the conformational change in the RsrA domain in RCP when it binds to SYP in a reducing environment or releases SYP in an oxidizing environment. The conformational change in the RsrA domain in RCP was confirmed by Far-UV circular dichroism (CD) (Fig. 2E). A significantly sloping baseline (often becoming increasingly negative towards lower wavelengths) may indicate that there is a disulfide contribution to the spectrum of purified RCP [33]. Characteristics of secondary structure calculated by CDNN indicate approximately 25% α-helices and 26% β-sheets in RCP, and 32% α-helices and 17% β-sheets in RCP oxidized with H_2_O_2_. Also, RCP presents a less helical and more β-sheet-like structure in a reduced condition with DTT, indicating that disulfide formation leads to a major conformational change in the whole recombinant protein. The results are similar to previously reported CD spectra of native RsrA which indicates that the recombinant proteins SYP and RCP exactly work as original proteins σ^R^ and RsrA when they response to redox environment [8]. To exclude interference from conformational changes in the CFP protein, CD spectra of both reduced and oxidized CFP were performed as a control, showing no difference between disulfide conformation characteristics (Fig. 2F).

### Using the FRET-based system to probe the protein-protein interaction between σ^R^ and RsrA

**Figure 3:**
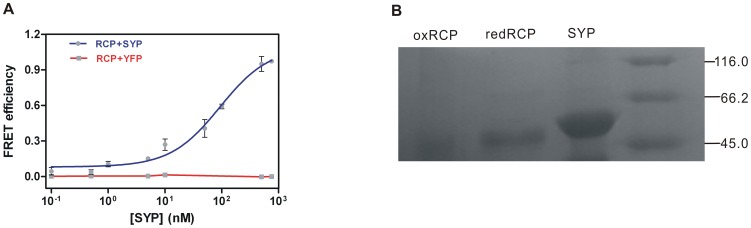
Thermodynamic binding curve of SYP and RCP. (A) Values of FRET efficiency in each group of samples were detected. The blue line presents the binding of RCP with increasing concentrations of SYP fitting a curve of single-site binding equation. The red line presents the binding of RCP with increasing concentrations of YFP as reference. Data represent the means ± SEM of three independent measurements. (B) Redox status of RCP and purity of the FRET-based probes were measured by non-reducing SDS-PAGE. Lines shows purified oxidized RCP (through exposed to air for 2 hours), reduced RCP (through dialysis in DTT containing binding buffer), purified SYP, the unstained protein molecular weight marker (Thermo Scientific).

RsrA binds to σ^R^ in a physiological or relatively reducing environment. However, means to directly research the protein-protein interaction between σ^R^ and RsrA are rare and limited. To further understand the interaction and binding properties of σ^R^ and RsrA, we used the pair of FRET-based probes to detect and evaluate their *Kd* constant (Fig. 3A). Since the FRET system consists of bimolecular probes, we used the FRET efficiency (E), as described in Materials and Methods, to discern the signal changes. It was detected by the binding between a predetermined concentration of donor RCP and increasing concentrations of acceptor SYP. The obtained values of E were fitted to a simple single-site binding model. The dissociation constant *Kd*, the ligand SYP concentration at half-maximal saturation, was calculated to be 81 ± 29 nM. DTT was included in binding buffer to ensure reduced RCP. The redox status of RCP is shown in Fig. 3B. In the case of the control, we added increasing concentrations of YFP to RCP under the same conditions but observed nearly null FRET efficiency, indicating no protein-protein interaction between RCP and YFP even though they both emit fluorescence. This FRET-based system blazes a new path for researching protein-protein interactions.

### FRET-based system verifies the role of zinc in the σ^R^-RsrA redox switch

**Figure 4:**
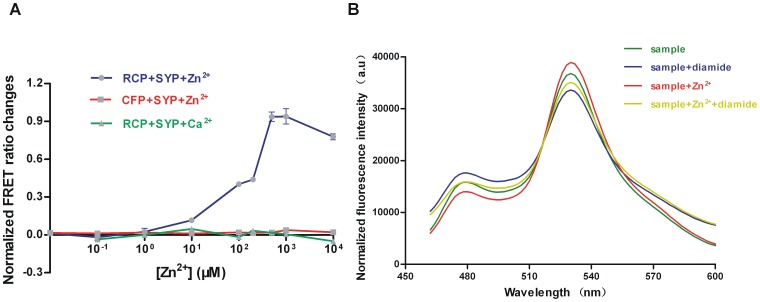
Effect of zinc on interaction of σ^R^ and RsrA. (A) Zinc effect on interaction of SYP and RCP. Blue line shows the effect of various concentrations of zinc ion on RCP when it binds to SYP, while the green line shows the effect of Calcium ion and the red line shows the the zinc effect on CFP mixed with SYP as control. Data represent the means ± SEM of three independent measurements. (B) Fluorescent emission spectra of probes in response to diamide with and without ZnSO_4_. Both Zn-sample and apo-sample without diamide treatment were detected as control and spectrum of each assay normalized to 520 nm.

To confirm the function of zinc in modulating the protein-protein interaction between σ^R^ and RsrA using the FRET-based probes, we firstly determined the concentration of added zinc ions at physiological levels because an overdose of metal ions could photobleach the fluorophores. From the data, the ΔFRET ratio increased gradually with the addition of concentrated ZnSO_4_ up to a final concentration of 1 mM, indicating that zinc enlarges the binding distance between SYP and RCP (Fig. 4A). However, the FRET ratio decreased after the addition of more than 1 mM ZnSO_4_. Mixtures of CFP and SYP were also treated with increasing concentrations of ZnSO_4_ and no FRET-on signal appeared. Also, no apparent FRET ratio changes were shown after addition of other type of ion like Ca^2+^. These results prove that zinc has no effect on CFP or the fused fluorescent protein but a large effect on the RsrA domain of RCP, leading to an enhanced FRET-on signal. High concentrations of ZnSO_4_, like 10 mM, may cause denatured protein, which renders a relatively negative effect on the interaction. Then we added 500 μM ZnSO_4_ to a predetermined same concentration of SYP and RCP, and compared the FRET signal from the fluorescence intensity spectrum (FRET ratio 2.8 for emission at 528 nm/478 nm) to that of a sample with no ZnSO_4_ added (FRET ratio 2.3) (Fig. 4B). Also, the sample with added Zn^2+^ was more stable and less oxidized when it responded to the same concentration of diamide. In the diamide treatment assays the values for the FRET ratios in the Zn-sample (2.2) and apo-sample (2.0) were different, which indicates their different extents of disulfide bonds or different disulfide bond formations. Zn-RCP forms intramolecular disulfide bonds and releases zinc ion when suffering relatively lower concentration of diamide, but for some extent it still binds to SYP (with lower affinity). The lowest FRET ratio presented by apo-RCP possibly suggests that further oxidation after zinc releasing causes relatively complete dissociation with SYP.

### FRET-based system for directly detecting the sensitivity of the σ^R^-RsrA complex to various ROS

**Figure 5:**
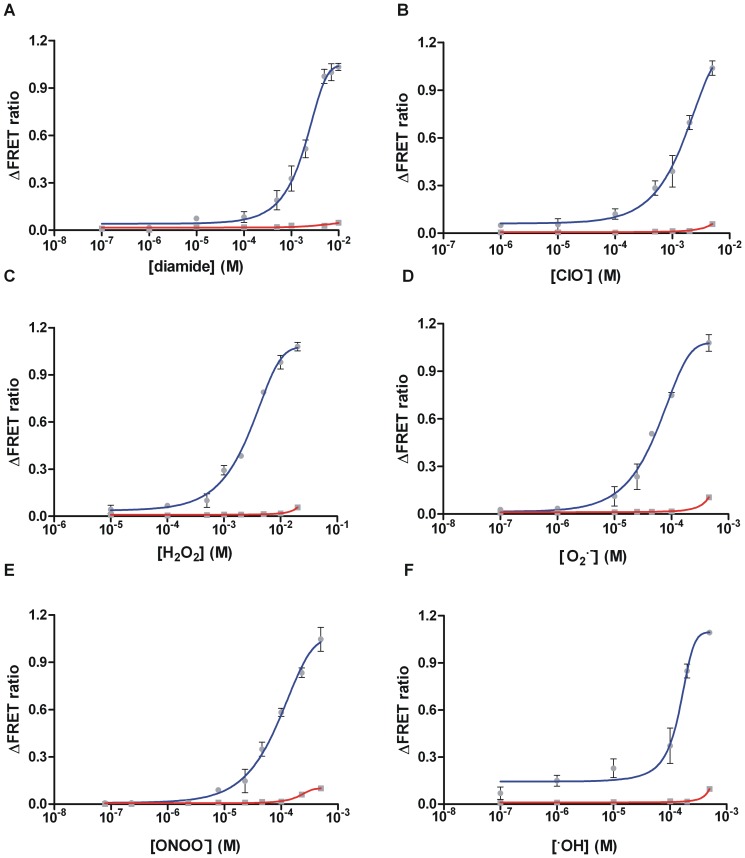
Dose-response curve for fluorescence detection of the FRET-based probes to various oxidants (A) diamide, (B) -OCl, (C) H2O2, (D) ·OH, (E) O2·- amd (F) ONOO- in increasing concentrations. Sample consisted of determined and the same concentration of RCP, SYP and Zn^2+^. The blue line in each assay presents the fitted curve for the related oxidant, and the red line presents the effect of each oxidant on the YFP and CFP mixture as control. Data represent the means ± SEM of three independent measurements.

Traditional methods of *in vitro* assays have demonstrated that oxidants like peroxide directly trigger σ^R^-RsrA redox switching through disulfide stress. In physiological situations, oxidative stress during metabolism turns on the switch by producing ROS from cellular activity. For example, as a consequence of oxygen being the ultimate electron acceptor in respiration, hydroxyl radicals (**^·^**OH) and superoxide free radicals (O_2_ **^·^** ^−^) are generated, which play important roles in the growth and secondary metabolism of *S. coelicolor* [34]. To further understand how ROS turns on the holistic redox switch and the regulatory system is initiated through conformational changes and protein-protein interactions, we determined the sensitivity of the (zinc containing) σ^R^-RsrA complex to diamide and various ROS from different metabolic pathways (Fig. 5). Compared to diamide, a thiol-specific oxidizing treatment as a reference, the changes in the ΔFRET ratio for each ROS assay proves that the disulfide formation in RsrA resulting in release of zinc and dissociation of σ^R^ can also be induced by ROS directly. However, the fitted dose-response curves of normalized ΔFRET ratio values show different sensitivities of the σ^R^-RsrA complex to various ROS, which may result from their different oxidative properties for RsrA. We found that the σ^R^-RsrA complex is most sensitive to **^·^**OH, O_2_ **^·^** ^−^ and peroxynitrite (ONOO^−^) (Fig. 5D-F), less sensitive to hypochlorite (^−^OCl) and least sensitive to peroxide (Fig. 5B-C). The curve of the O_2_ **^·^** ^−^ group showed the highest sensitivity (Fig. 5D) compared to other ROS and the diamide group. It has been reported that ONOO^−^ oxidizes sulfhydryl groups 1000 times faster than peroxide [30]. The slope of the ONOO^−^ curve is similar to that of diamide but more sensitive, indicating its thiol specific and stronger oxidative nature (Fig. 5E). The curve for the **^·^**OH group also presents a strong oxidizing property with similar but slightly less sensitivity compared to that of ONOO^−^ (Fig. 5F). However, the σ^R^-RsrA complex presented almost two orders of magnitude of less sensitivity to ^−^OCl and peroxide than to other ROS, even though ^−^OCl has strong oxidizing potential. In the case of diamide (Fig. 5A), the ΔFRET ratio was saturated after the addition of an overdose (greater than 5 mM) of the oxidant. But the ΔFRET ratio still changed because of the large effect of the oxidative properties of various ROS on the fluorophores. Therefore, we determined the effect of increasing concentrations of various ROS on the same concentrations of YFP and CFP mixture (including 500 μM Zn^2+^) as control. Only very high concentration of each ROS presented obvious effects on the fluorophores.

### Application of an RsrA based biosensor to detect the redox environment

**Figure 6:**
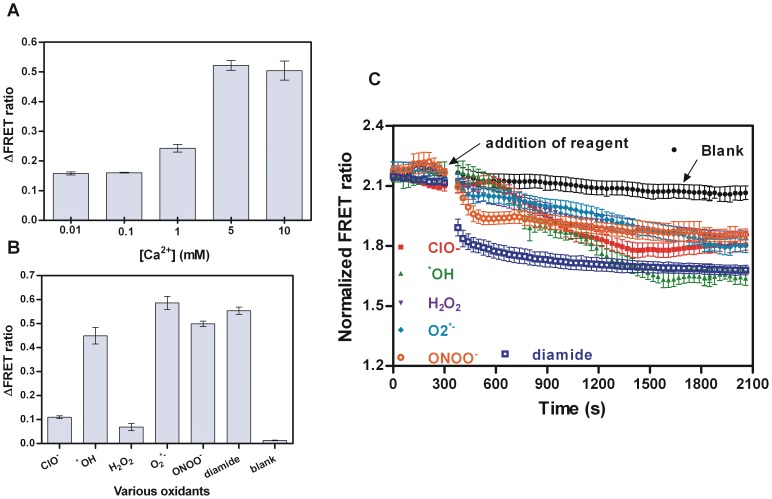
Application of RsrA based biosensor detecting redox environment. (A) Effect of Ca^2+^ on sensitivity of of BL21 to high concentrations of H_2_O_2_. The increasing concentrations of Ca^2+^ as shown on graph were incubated with sensor and the values of ΔFRET ratio were obtained. (B) Sensor containing BL21 in response to the same concentration of various ROS as described in Materials and Methods. The ΔFRET ratio of each oxidant (shown on graph) was obtained. All data represent the means ± SEM of three independent measurements. (C) Time course of sensor monitoring changes in redox status induced by various ROS in BL21. Addition of appropriate concentration of ROS is indicative at 5 minute time point. The addition operation interrupted the measurement for approximate 7 s. The black points present the reference with addition of buffer. The data from each assay was normalized.

Presently, the most promising tools for non-invasive, specific, dynamic, *in-situ* and compartment-targeted detection are genetically-encoded fluorescent probes [35], [36]. The σ^R^-RsrA switch-based FRET system developed in this study can also be employed to monitor dynamic redox changes induced by ROS in living cells during respiratory or nitrogen source metabolism [37], [38]. The *E. coli* BL21 (DE3) cells that expressed SYP and RCP were employed for the assays. We found that CaCl_2_ treated bacteria (competent cells) were more sensitive to oxidants. To extend the ROS and diamide response we treated the bacteria with calcium ions (Ca^2+^) to render them competent and noted increasing concentrations of Ca^2+^ presented a positive effect on the ΔFRET ratio (Fig. 6A). As the Ca^2+^ concentration increased the value of the ΔFRET ratio increased and the optimized concentration of Ca^2+^ (5 mM) was chosen for further assays. The ΔFRET ratio of probe-containing *E. coli* BL21 (DE3) cells was then scanned after the addition of the same concentration of various ROS and diamide (100 μM) (Fig. 6B). The differences between each obtained ΔFRET ratio indicate the different sensitivities and selectivities of the probes to different oxidants. To monitor the process of diamide (disulfide stress) and ROS (oxidative stress) treatment *in vivo* by means of this pair of redox switch based biosensors, FRET emission ratios (528 nm/478 nm) in the *E. coli* BL21 (DE3) cells were measured over time, and in each assay after 5 minutes of reaction a corresponding ROS or diamide was added (Fig. 6C). The concentration of ROS or diamide must be determined carefully because an overdose during the experiment would be lethal to the bacteria. We chose various ROS whose concentration at half-maximal saturation of ΔFRET ratio in each curve of *in vitro* dose-response experiment and 1 mM diamide to monitor the biosensor containing bacteria. As expected, the decreases in the FRET ratio over time were in agreement with the dose-response experiment *in vitro*, and the saturation endpoints tended to be similar except in the O_2_ **^·^** ^−^ assay. A lower saturation point in the FRET ratio may result from cell damage due to oxidative stress. Thus the released free probes may be reacting with O_2_ **^·^** ^−^ directly which led to a higher FRET-off signal. On the other hand, although the *in vivo* test showed a general response to a specific ROS it may also be affected by trace amounts of byproduct; herein O_2_ **^·^** ^−^ could dismutate to form H_2_O_2_, which possibly oxidized intracellular iron to generate small amounts of **^·^**OH, which may also lead to a higher FRET-off signal. As expected, the results of diamide treatment showed as sensitive and complete a response reaction as disulfide stress. Therefore, this data suggests that during the growth of *S. coelicolor*, especially in the stationary phase, ROS at physiological concentrations turning on the redox switch is based on overlapping effects. The decrease with **^·^**OH treatment was even smoother than that with H_2_O_2_ treatment, which probably derived from the lag of Fenton reactions which produce **^·^**OH. Compared to the control blank, the saturation FRET ratio of ROS samples during the last 10 minutes tended to rise back slightly, possibly indicating neutralization of a portion of the added ROS by the anti-oxidative system of *E.coli* and reversible interaction between σ^R^ and RsrA in living *E. coli* cells. The slow recovery could be the result of a cellular response, likely peroxide-sensing OxyR of *E. coli* itself, to a change in cellular redox status.

## Discussion

It has been reported that zinc plays a crucial role in stabilizing the conformation of RsrA and modulating its redox activity. However, the model for the single zinc site in RsrA and the intramolecular disulfide bond formation remain to be determined. Li *et al.* proposed a model that presents the conserved HX_3_CX_2_C (HCC) motif His37, Cys41, and Cys44 with an additional Cys11 to be the metal ligands, and the disulfide between Cys11 and Cys44 to be the result of oxidized RsrA [8], while Bae *et al*. provided a model that predicts Cys3, His7, Cys41, and Cys44 to be the metal ligands, and an extra disulfide between Cys41 and Cys61 within oxidized RsrA [13]. In addition, Zdanowski *et al.* utilized random and sit-specific mutagenesis with zinc K-edge extended X-ray absorption fine structure (EXAFS) spectroscopy to prove zinc site in RsrA to be Cys11, His37, Cys41, and Cys44, and trigger disulfide between Cys11 and Cys44 which results in the release of zinc during oxidation [18]. Their EXAFS data also show different redox status of RsrA during oxidation, and identify that formed disulfide can oxidize other cysteines in RsrA through thiol-disulfide exchange during further oxidation, which might support the model that Bae *et al*. proposed. In our FRET-based data, since σ^R^ and RsrA perform the function of SYP and RCP respectively, these results not only draw an agreement with previous studies, but suggest the difference between Zn-RsrA and apo-RsrA. The stronger FRET-on signal in the presence of a zinc ion suggests that zinc can stabilize the structure of RsrA (in RCP) when binding to σ^R^ (in SYP), and the binding of apo-RsrA (without zinc) to σ^R^ would be weaker compared to that of Zn-RsrA. During oxidation and relying on a more compact structure of zinc ligands, Zn-RsrA might form a more regular and lesser extent disulfide bond, whereas apo-RsrA might preferentially form different or more disulfide bonds which facilitate to the dissociation with σ^R^. Therefore, the whole response process of RsrA probably should not be one step reaction [18], but a trigger of disulfide formation leading to simultaneous release of zinc followed by further disulfide bonds which cause fully oxidized RsrA and dissociation with σ^R^. We propose the hypothesis that the redox switch forms an apo-RsrA intermediate during oxidative response, which may interpret why Zn-RCP and apo-RCP show different FRET signal after incubation with the same concentration of diamide.

ROS are chemically reactive molecules containing oxygen and result in macromolecular damage in cells. Cellular ROS sensing and metabolism are tightly regulated by a variety of proteins to maintain cellular redox homeostasis. Sensitive and selective detection probes for cellular redox status should be powerful tools for studying signal transduction, oxidative modification of proteins, and the stress response. So far, many fluorescent sensor probes have been widely used for imaging ROS, GSH, and redox status in cells, including chemical fluorescent dye probes [10], [39] and genetically encoded fluorescent probes [40]-[42]. Chemical fluorescent probes can cause toxicity and perturb cell metabolism. FRET-based genetically encoded biosensor can be readily used in probing protein-protein interactions, protein conformational changes and kinetics [43], [44]. In this study, we use FRET-based genetically-encoded biosensor not only to probe the protein-protein interaction between σ^R^ and RsrA in *S. coelicolor* in response to redox environment, but also to detect changes in redox status induced by various ROS in bacteria in real time. From the FRET signal we can identify the redox status of RsrA which determines its distance to σ^R^. Since the redox status of RsrA is determined by redox environment, the amount of oxidants or ROS can be detected through the change of FRET signal. However, applications to detection as a biosensor require focusing on sensitivity, selectivity and repeatability, and the bimolecular sensor carries disadvantages [45]. Thus, based on the modified RsrA, an optimized redox sensitive peptide fused to a fluorophore as a monomolecular biosensor remains to be developed for monitoring the redox environment.

ZAS proteins, belonging to a family of anti-sigma factors, are encoded next to Group IV ECF sigma factor genes and are characterized by a conserved ZAS motif (HxxxCxxC). These ZAS-related anti-sigma factors are very widespread in both Gram-positive and Gram-negative bacteria, playing an important role in transcriptional regulation responding to a variety of different stresses [46], [47]. The FRET-based system in this study provides a novel idea to design powerful tools for investigating the signal-sensing functions of other reported or predicted relevant ZAS family factors (like RshA from *Mycobacterium tuberculosis*, RsiW from *Bacillus subtilis,* ChrR from *Rhodobacter sphaeroides,* etc.) on the stress responses.
